# Early High-Grade Thoracic Toxicity After Palliative Radiotherapy for Non-Small Cell Lung Cancer

**DOI:** 10.7759/cureus.12494

**Published:** 2021-01-05

**Authors:** Carsten Nieder, Kristian Imingen

**Affiliations:** 1 Oncology, Nordland Hospital Trust, Bodø, NOR

**Keywords:** lung cancer, radiotherapy, chemoradiation, palliative radiation therapy, hospitalization, toxicity

## Abstract

Introduction: Palliative radiotherapy or chemoradiotherapy (CRT) for non-small cell lung cancer (NSCLC) may cause thoracic toxicities due to the radiation dose delivered to the lungs, heart, and esophagus. We studied severe thoracic toxicities resulting in hospitalization or death during the acute and sub-acute phase, i.e., three months from commencing radiotherapy. In addition, risk factors were identified.

Methods: A retrospective review of 165 patients treated with three-dimensional conformal palliative radiotherapy or CRT was performed. The prescribed total dose was equivalent to at least 30 Gy in 10 fractions. Uni- and multivariate analyses were employed.

Results: Twelve patients (7%) were hospitalized within three months from the start of radiotherapy or CRT. Six patients were hospitalized for esophagitis, three for dyspnea most likely caused by pneumonitis, and three for cardiac arrhythmia. Fatal toxicity was not observed. However, 19% of the 165 patients died from tumor-related causes during the time period of interest. In multivariate analysis, the only esophageal dose was significantly associated with the risk of hospitalization.

Conclusion: The safety profile of palliative radiotherapy or CRT in the acute and subacute phases was satisfactory. The hospitalization rate can be reduced by lowering the esophageal dose, as long as safe lung and heart doses can be maintained.

## Introduction

Despite the well-proven and highly relevant clinical benefits of palliative radiotherapy for non-small cell lung cancer (NSCLC), this treatment frequently induces acute and sub-acute side effects, e.g., esophagitis, weight loss, and fatigue [1-3]. Especially with total doses equivalent to at least 30 Gy in 10 fractions and combination with chemotherapy, pneumonitis, and cardiac adverse events might also be observed, though not to the same degree as in the radical treatment setting [4-7]. Intermediate radiation doses between 30 and 60 Gy, such as the Norwegian CONRAD regime (42 Gy in 15 fractions), are also endorsed in current guidelines, preferably in combination with platinum-based chemotherapy [8,9]. Both chemotherapy and radiation are highly likely to induce at least mild or moderate side effects [10,11]. Acute and sub-acute thoracic toxicities causing hospitalization or death are of particular concern in a palliative treatment setting, where the expected outcome is symptom improvement and/or prolongation of survival. In order to study treatment safety, we performed a retrospective analysis of hospitalization and death within three months from the start of palliative radio- or chemoradiotherapy.

## Materials and methods

The authors’ institution treated 165 consecutive patients with palliative three-dimensional conformal radiotherapy or chemoradiotherapy to an equivalent dose of at least 30 Gy in 10 fractions between 2009 and 2019. These patients were included in the study, whereas those treated with low palliative doses, primarily two fractions of 8.5 Gy, were excluded. In case of chemoradiation, most patients received the Norwegian CONRAD regime (15 fractions of 2.8 Gy, four cycles of carboplatin/vinorelbine before and during radiotherapy) [8]. Clinical information throughout follow-up was abstracted from the hospital's electronic patient record system in order to capture hospitalization after the start of radiotherapy. The system also captures hospitalization at all other hospitals in our healthcare region, thus providing complete data. This aspect is important because many patients live remote from the region’s main hospital, which provides all radiotherapy. The acute and sub-acute phase was defined as the first three months after the start of radiotherapy. After the first follow-up visit at 6-8 weeks from the final day of radiotherapy, intervals were increased to three months. Side effects were graded according to the common terminology criteria for adverse events (CTCAE) version 4.0. There was no consistent recording of symptom relief (pain, cough, etc.) and therefore, this endpoint was not evaluated. Treatment plans were calculated with Varian Eclipse TPS® (Varian Medical Systems, Palo Alto, CA) and no intensity-modulated or arc-based techniques were employed. Dose-volume histograms were accessed to abstract dosimetric variables, e.g., heart, lung (both lungs combined minus clinical target volume {CTV}), and esophageal equivalent dose (EQD2) that might correlate with the risk of side effects. IBM Statistical Package For The Social Sciences (SPSS®) v.25 (IBM Corp., Armonk, NY) was employed for the statistical analyses. The latter included chi-square test and binary logistic regression for associations between thoracic toxicity causing hospitalization or death (present/absent) and clinical and dosimetric variables. Significant variables, i.e., p<0.05 in two-sided tests, were then included in a multi-nominal logistic regression analysis. Actuarial overall survival was calculated according to the Kaplan-Meier method. Log-rank tests were used to compare the actuarial survival curves.

## Results

The median age was 69.5 years, range 41-90. Ninety patients (55%) were men. Stage distribution was as follows: I and II in 6%, III in 46%, IV in 48%. The histology was adenocarcinoma in 40% and squamous cell carcinoma in 40% (other or unspecified in 20%). The median size of the CTV was 134.5 ml, range 10-1185. Thirty-six percent had a diagnosis of chronic obstructive pulmonary disease (COPD). Thirty-three percent were treated with 10 fractions of 3 Gy (“low” dose), 20% with “intermediate” dose (e.g., 13 fractions of 3 Gy), and 47% with “high” dose (e.g., 15 fractions of 2.8 or 3 Gy). Concomitant chemoradiotherapy was given in 32%.

**Figure 1:**
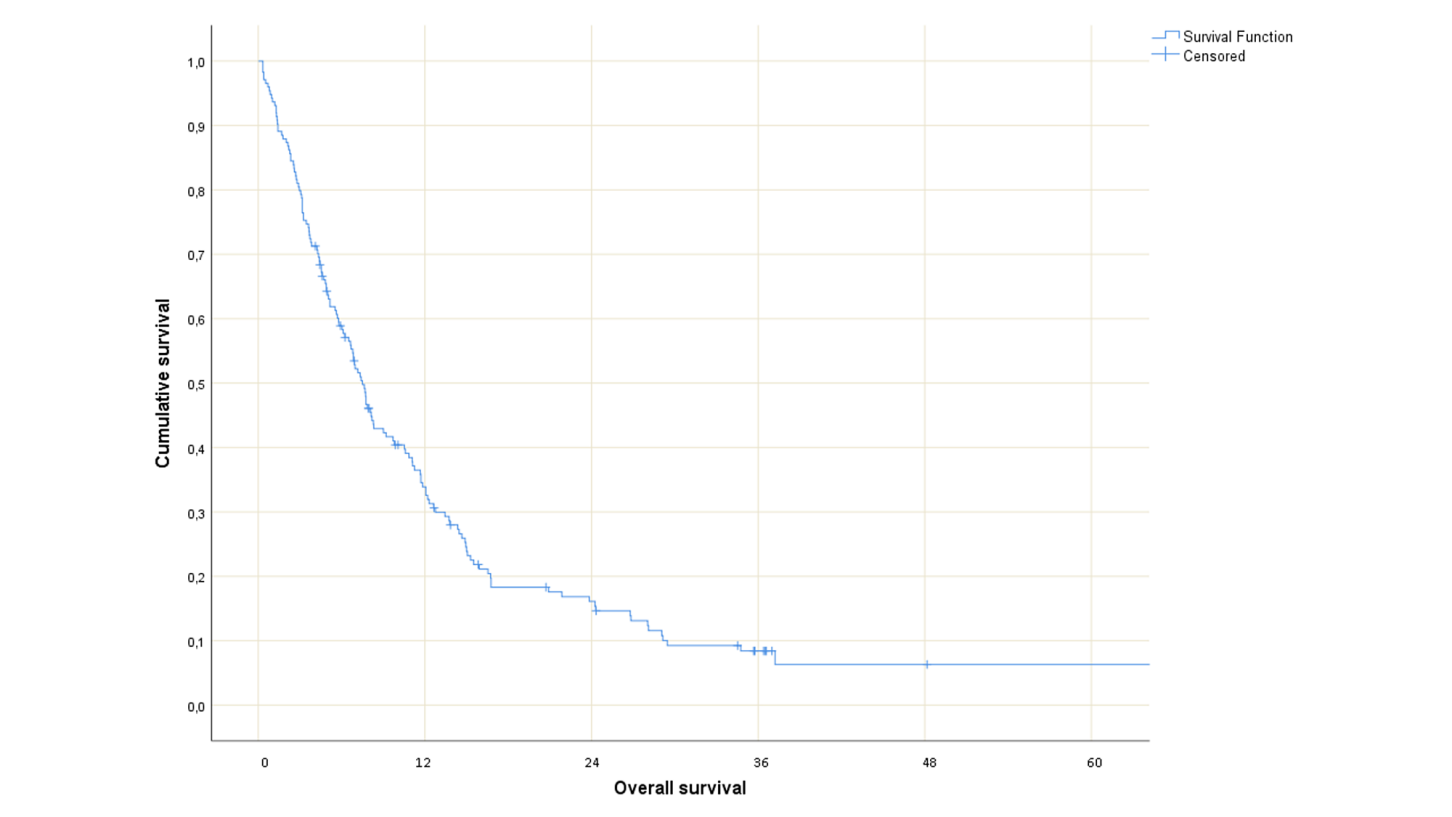
Actuarial overall survival in months (Kaplan-Meier curve) for all 165 patients

At the time of this analysis, 18 patients were alive (censored observations after a median follow-up of 14.4 months, a minimum of four months). The date of death was known for all remaining patients. Thirty-two patients (19%) died during the three-month time period of interest (Figure 1).

**Figure 2:**
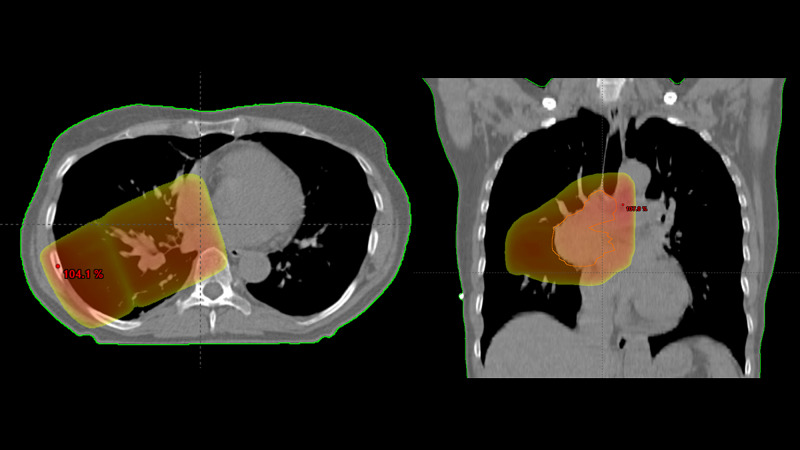
Representative axial and coronal treatment planning computed tomography scan showing the 80% isodose color wash in a patient treated for right-sided T2 N2 disease (15 fractions of 2.8 Gy) who was hospitalized for cardiac arrhythmia The maximum dose is displayed in red. The orange line depicts the clinical target volume.

None of these deaths was related to toxicity. Twelve patients (7%) were hospitalized for thoracic toxicity during the same time period (six for esophagitis CTCAE grade 3, three for dyspnea most likely caused by pneumonitis (CTCAE grade 3), and three for cardiac arrhythmia). In the absence of toxicity-related deaths, predictive factors for hospitalization were evaluated. As shown in Table 1, dosimetric variables correlated with this endpoint, in particular esophageal and heart dose (Figure 2). 

**Table 1:** Factors associated with hospitalization for thoracic complications during the first three months (univariate analyses) EQD2: equivalent dose in 2-Gy fractions (alpha/beta value 2 Gy for heart and 10 Gy for esophagus) Other parameters with p-value >0.1: chronic obstructive pulmonary disease, active smoking, sex, location left vs. right lung, T stage, N stage, lung cancer stage (II, III, IV). Patients hospitalized for esophagitis had a median esophageal maximum EQD2 of 46.5 Gy (median mean esophageal EQD2 was 28.1 Gy).

Parameter	Hospitalized	Not hospitalized	Significance level
Median heart EQD2 (maximum dose to 1cc), Gy	36.3	25.1	0.027
Median lung volume treated to 20 Gy (V20), %	25	19	0.16
Median mean lung dose (MLD), Gy	11.9	9.7	0.12
Median mean esophageal EQD2, Gy	23.5	13.8	0.005
Median esophageal maximum EQD2, Gy	46.5	40.6	0.024
Median clinical target volume (CTV), cc	113	135	0.59
Median planning target volume (PTV), cc	402	401	0.80
Median age, years	73	69	0.31
High radiation dose, %	12	88	
Low or intermediate radiation dose, %	3	97	0.044
Concomitant chemoradiotherapy, %	19	81	
No concomitant chemoradiotherapy, %	4	96	0.01

Higher prescribed dose and concomitant chemotherapy were also associated with the risk of hospitalization, in contrast to other factors such as age, sex, COPD, smoking status, overall stage, or side of the primary tumor. The multi-nominal logistic regression analysis showed that mean esophageal dose was the dominant factor predicting for hospitalization (p=0.034), while concomitant chemotherapy and the other variables were not significant (Table 2).

**Table 2:** Multi-nominal logistic regression analysis The variables were dichotomized (yes/no or by median), except for radiation dose (three strata).

Hospitalization		B	Std. Error	Wald	Df	Sig.	Exp(B)	95% Confidence interval for Exp(B)	
								Lower bound	Upper bound
	Intercept	1,009	,496	4,143	1	,042			
	Concurrent chemoradiotherapy =0	,955	,742	1,655	1	,198	2,598	,606	11,133
	Concurrent chemoradiotherapy =1	0^b^	.	.	0	.	.	.	.
	Radiation dose=0	-,081	1,534	,003	1	,958	,922	,046	18,658
	Radiation dose=1	,432	1,294	,112	1	,738	1,541	,122	19,448
	Radiation dose=2	0^b^	.	.	0	.	.	.	.
	Dmax heart=0	,555	,739	,564	1	,453	1,742	,409	7,417
	Dmax heart=1	0^b^	.	.	0	.	.	.	.
	Dmean esophagus=0	2,119	1,149	3,403	1	,034	8,322	,876	79,058
	Dmean esophagus=1	0^b^	.	.	0	.	.	.	.
	Dmax esophagus=0	,265	1,201	,049	1	,825	1,303	,124	13,718
	Dmax esophagus=1	0^b^	.	.	0	.	.	.	.

**Figure 3:**
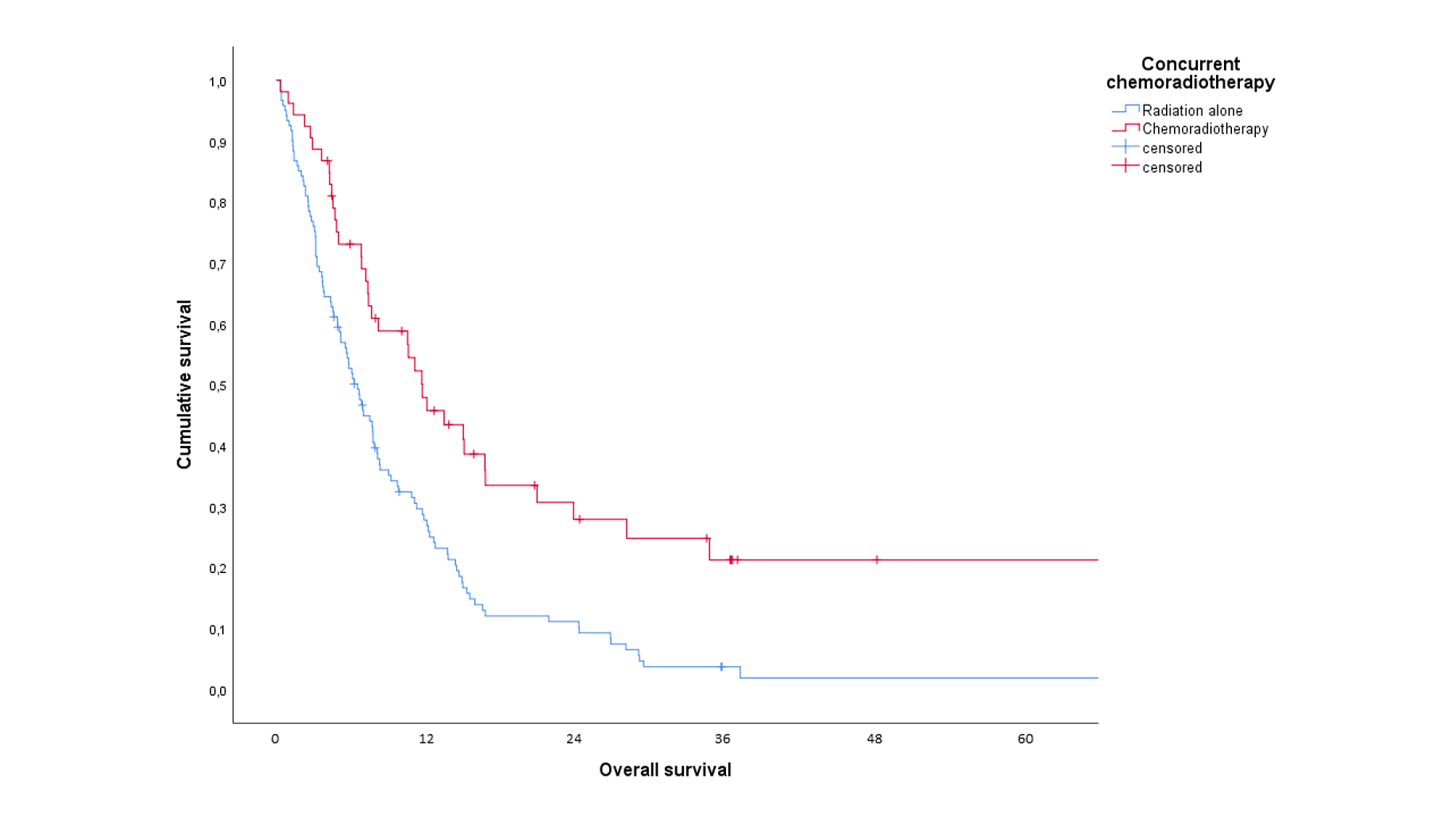
Actuarial overall survival in months (Kaplan-Meier curve) stratified by treatment

There was no significant difference in actuarial overall survival between the two groups (median 10.5 months if hospitalized due to toxicity versus 7.6 months if not, p=0.26). In contrast, patients who were treated with concomitant chemotherapy survived significantly longer (median 11.7 months versus 6.5 months with radiotherapy only, p=0.0001, Figure 3).

**Figure 4:**
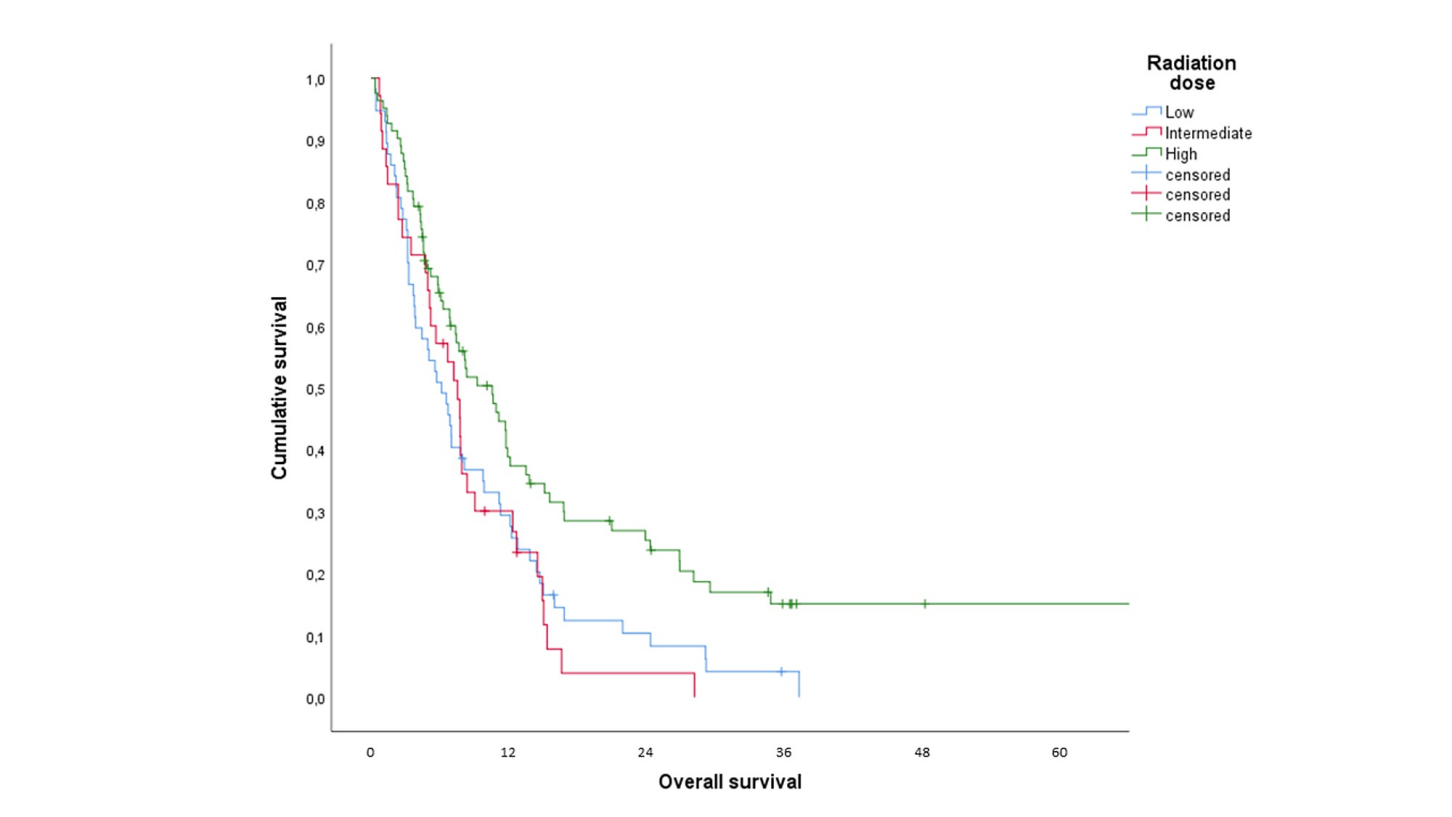
Actuarial overall survival in months (Kaplan-Meier curve) stratified by radiation dose

High radiation dose was also associated with better survival (median 10.5 months versus 7.5 {intermediate} and 6.1 months {low}, p=0.007 {pooled over all strata}, Figure 4).

## Discussion

This retrospective analysis focused on toxicity-related hospitalization and death within three months from the start of palliative radio- or chemoradiotherapy in 165 patients with NSCLC. During this time period, maintenance chemotherapy or tyrosine kinase inhibitors were not used. It was reassuring to see that only 7% of the patients were hospitalized and that none of the toxicities resulted in death. Disease progression caused death in the time period of interest is 19% of the patients, a fact that is understandable because 48% had stage IV NSCLC. The “two fractions of 8.5 Gy”-regime or other short-course radiotherapy regimes might have been a good choice for symptom palliation in these patients with short survival [12-14]. We have previously proposed a prognostic model that might help assess these patients before prescribing a 10-fractions or more regime [15]. Performance status, serum lactate dehydrogenase, C-reactive protein, presence of liver/adrenal gland metastases, and extrathoracic disease status significantly predicted survival and formed the basis of the score. Early palliative and supportive care rather than palliative radiotherapy may also be appropriate for patients with many adverse prognostic features [16]. Depending on prognosis, the goal of treatment, target volume size, and lung dose, the authors continue to prescribe 10 or 13 fractions of 3 Gy or, together with Carboplatin/Vinorelbine, 15 fractions of 2.8 Gy. 

The main toxicity identified in our patients was esophagitis (CTCAE grade 3). Cardiac arrhythmia and dyspnea might have multifactorial or unrelated causes [17-19]. However, the clinicians at the study site judged the respective hospitalizations as most likely treatment-related after careful consideration of other differential diagnoses. Given that esophagitis caused 50% of the hospitalizations, it was not surprising that the multi-nominal regression analysis identified esophageal dose as the only significant factor predicting hospitalization. Age, sex, and other patient- or tumor-related variables were not statistically significant. The duration of hospitalization varied (minimum two, maximum 16, median eight days). Standard supportive measures such as analgesics and parenteral nutrition were initiated to manage esophageal toxicity. We have previously published esophageal dose constraints for palliative (chemo)radiotherapy, based on a smaller study [10]. As mentioned in that study, our current treatment planning strategy is to limit the maximum dose to the esophagus (Dmax), e.g., by accepting a planning target volume (PTV) coverage of <95% at the intersection with the esophagus. If a high Dmax is unavoidable, we try to reduce the mean dose. The present univariate analysis also suggests that cardiac dose might be a parameter to study in a larger database with more high-grade toxicity events. It should also be noted that cardiac and pulmonary toxicities continue to manifest after longer time intervals than the three months studied here. Furthermore, grade 2 side effects, including esophagitis and pneumonitis, commonly deteriorate the patient-reported quality of life and should not be neglected [20,21]. 

In a pivotal Norwegian randomized study of chemotherapy versus chemoradiotherapy (42 Gy in 15 fractions, CONRAD), which showed the superiority of the combined approach regarding overall survival, 40% of the patients in the combined arm were hospitalized once and 11% twice in relation to side effects [8]. Many hospitalizations were caused by esophagitis. The trial protocol did not recommend specific dose constraints for this organ at risk. Some hospitalizations were due to chemotherapy-related toxicity, e.g., neutropenic infections. 

Despite inherent limitations of the retrospective study design, and the limited number of events, which reduces the statistical power, the study’s main strength should also be considered, i.e., complete baseline and follow-up data due to the availability of a comprehensive regional electronic patient record, and the equal-access-to-care setting guaranteed by the Norwegian healthcare system, which prevents financial barriers to hospitalization. 

## Conclusions

The safety profile of palliative radiotherapy or chemoradiotherapy in the acute and subacute phases was satisfactory. The hospitalization rate can be reduced by lowering the esophageal dose, as long as safe lung and heart doses can be maintained. Given that survival was longer after higher doses of radiation and also after concomitant chemotherapy, and that hospitalized patients had numerically longer survival, the added toxicity of more intense treatment may be acceptable. However, these aspects should be discussed with the patients during the initial consultation. Unfortunately, it was not possible to evaluate the palliative effect or symptom-relief of the treatment. 
